# Effectiveness of Enfortumab-Vedotin for Right Atrial Metastasis Following Total Cystectomy: A Case Report

**DOI:** 10.7759/cureus.75122

**Published:** 2024-12-04

**Authors:** Satoshi Tokitaka, Hiroshi Hirata, Reiko Shimo, Oka Shintaro, Koji Shiraishi

**Affiliations:** 1 Department of Urology, Graduate School of Medicine, Yamaguchi University, Ube, JPN

**Keywords:** atrial metastasis, bladder cancer, complete response, enfortumab-vedotin, pembrolizumab

## Abstract

Cardiac metastases from bladder cancer are extremely rare and typically associated with a poor prognosis. We here report a case of a 74-year-old woman who had been diagnosed with multiple bladder cancer and later developed pelvic recurrence and multiple bone metastases. Second-line pembrolizumab treatment achieved complete remission. Three years and three months after the second-line pembrolizumab treatment, a routine computed tomography scan revealed a right atrial mass and bilaterally enlarged adrenal glands. Primary tumors were not identified, suggesting that the tumor in the right atrium and the enlargement of the adrenal glands were recurrences of the previous bladder cancer. Enfortumab-vedotin (EV) was chosen as a subsequent treatment. One month after this treatment, the tumor in the right atrium was undetectable and her adrenal glands had shrunk. Her complete response was maintained six months after starting subsequent treatment. To the best of our knowledge, this is the first case report of complete remission of bladder cancer metastasis to the right atrium induced by EV.

## Introduction

Cardiac metastases, with an estimated incidence of 1.7%-14% across all cancer types, are rare. In particular, cardiac metastases originating from bladder cancer are extremely rare and typically associated with a poor prognosis [1]. Most patients with cardiac metastases from bladder cancer are asymptomatic, with their conditions often discovered incidentally during routine examinations. Chemotherapy remains the standard treatment for metastatic bladder cancer, typically followed by maintenance therapy with avelumab or pembrolizumab based on the response. Enfortumab-vedotin (EV) is generally administered as a subsequent treatment. Therefore, chemotherapy is likely the preferred option for managing cardiac metastases from bladder cancer. To our knowledge, there are no reported cases of cardiac metastases from bladder cancer being treated with EV, nor of complete responses (CRs) being maintained with this approach.

## Case presentation

**Figure 1:**
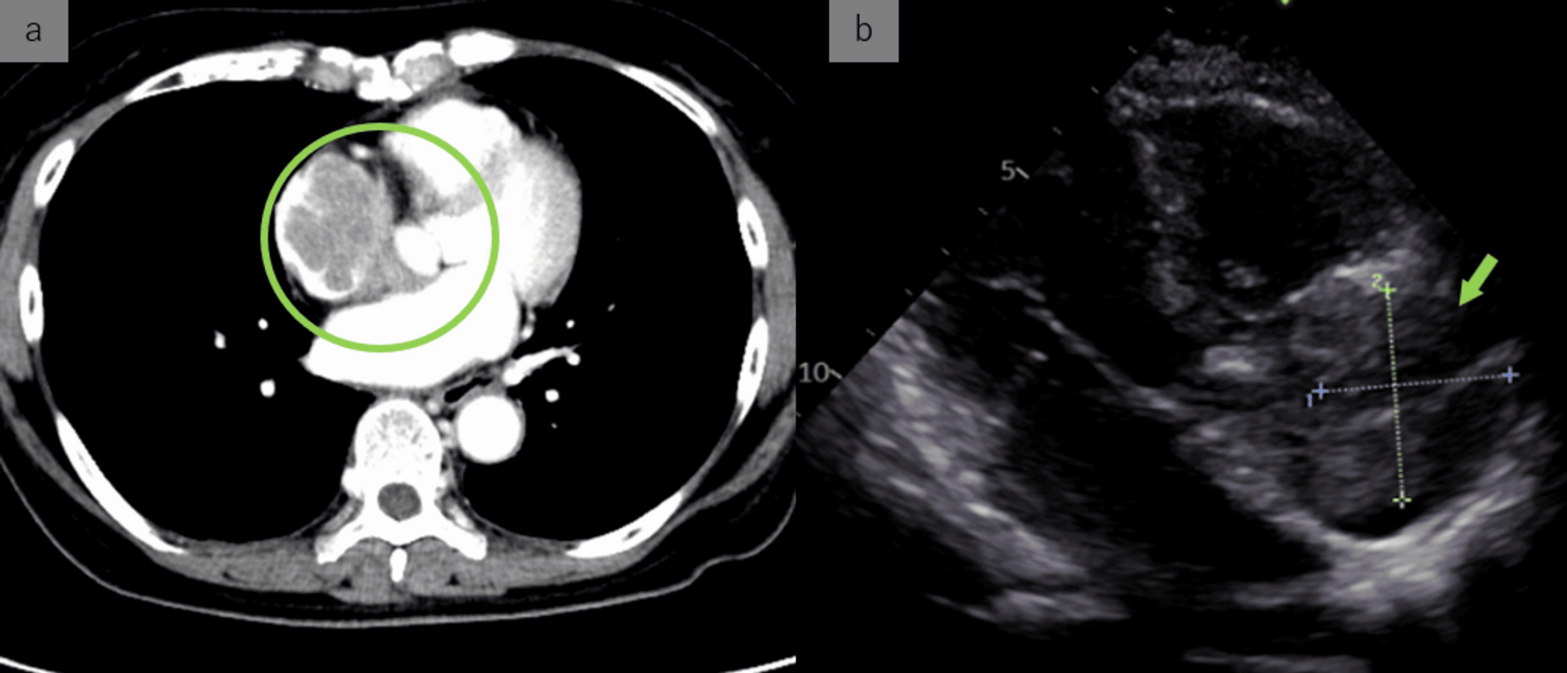
CT scan and echocardiography (a) A routine CT scan during continued pembrolizumab, revealed a contrast defect due to a mass occupying the right atrium. (b) Echocardiography showed the mass occupying the right atrium to be 40 x 33 mm in size.

The patient was a 74-year-old woman with no remarkable medical history prior to her presentation with gross hematuria. She was diagnosed with multiple bladder cancers and referred to our department for treatment. Her widespread papillary tumor extending from the bladder neck to the posterior wall was diagnosed by trans-urethral resection as invasive urothelial carcinoma, high grade, G3, pT2N0M0. Urine cytology was Class V. Two cycles of cisplatin-gemcitabine therapy were administered with total cystectomy in mind; however, bacillus Calmette-Guérin therapy was chosen with the hope of preserving the bladder. Later, intravesical recurrence prompted a laparoscopic total cystectomy. The planned surgery was achieved without any complications during or after the procedure. The histopathological diagnosis was pT0 in the bladder, carcinoma in situ in the right lower ureter, and RM0. Pelvic recurrence and multiple bone metastases were diagnosed approximately one year later, prompting the introduction of pembrolizumab as a second-line treatment. This treatment was continued without adverse events and achieved a CR. Three years and three months after the introduction of pembrolizumab, a routine computed tomography (CT) scan revealed bilaterally enlarged adrenal glands. Re-examination two months later revealed a mass occupying the right atrium and a further increase in the size of both adrenals. Echocardiography showed a 40 × 33 mm mass attached to the anterior wall of the right atrium. The ejection fraction was 60% (Figures 1a, 1b). At the same time, the patient complained of palpitations, and an electrocardiogram revealed ventricular tachycardia.

**Figure 2:**
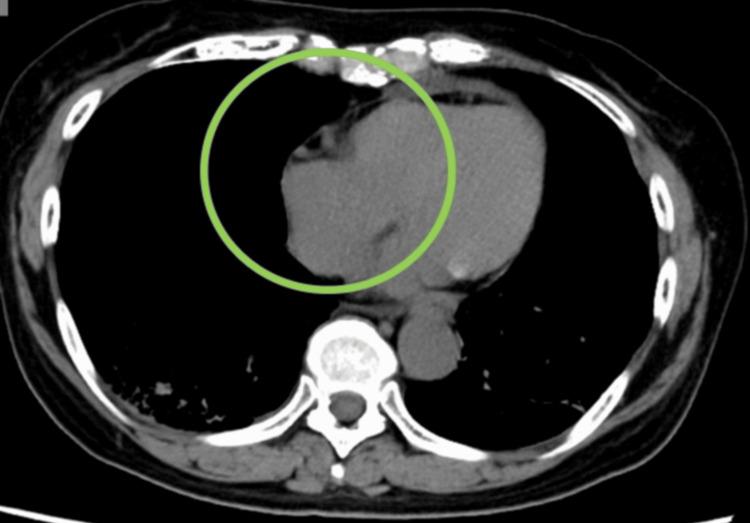
A CT scan taken three months later confirmed the disappearance of the right atrial tumor

**Figure 3:**
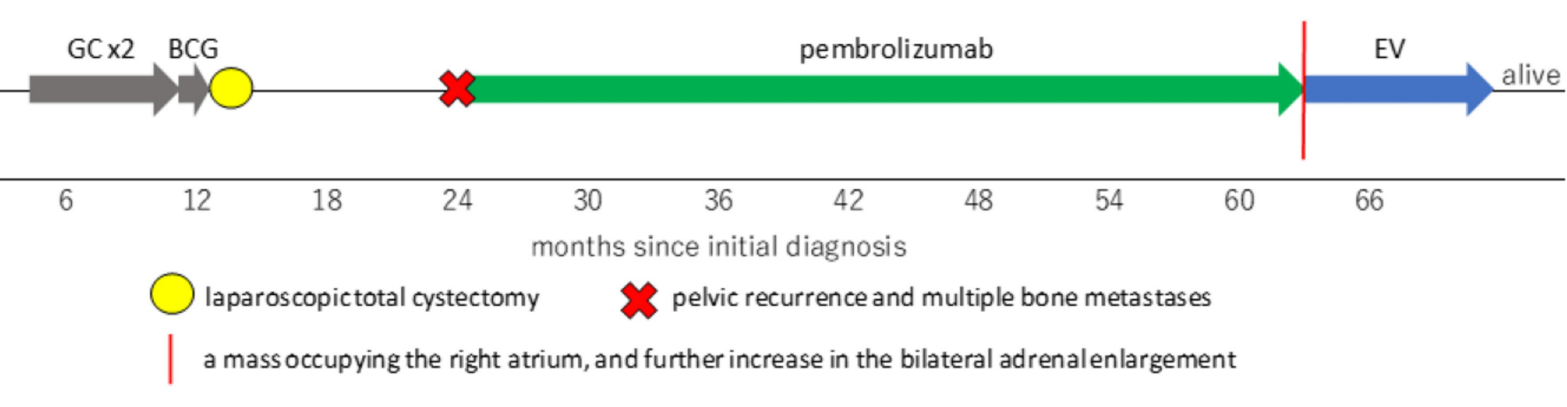
A swimmers’ plot Initially, two courses of adjuvant GC therapy were performed, BCG therapy was chosen with the hope of preserving the bladder, and in the end, laparoscopic total cystectomy was performed. For pelvic recurrence and multiple bone metastasis, pembrolizumab was introduced as a second-line treatment. It worked well for three years and three months. Because of a mass occupying the right atrium and a further increase in the bilateral adrenal enlargement, EV treatment was initiated as a subsequent therapy. GC - Glucocorticoids, BCG - Bacillus Calmette–Guérin, EV - Enfortumab-vedotin

The provisional diagnosis was recurrence of the bladder tumor in the right atrium and both adrenal glands. As it would have been difficult to make a histological diagnosis, EV treatment was initiated as subsequent therapy at 1.25mg/kg, according to the manufacturer's recommended regimen. Adverse effects included Grade 3 neutropenia, Grade 2 low white blood cell count, Grade 1 anemia along with thrombocytopenia, liver dysfunction, and drug-induced skin disorder. None of these adverse effects necessitated interruptions to treatment. One month later, at the end of the first cycle, a CT scan showed that the tumor in the right atrium had shrunk significantly, as had the adrenal glands, leading to a diagnosis of partial response. Furthermore, CT scan taken three months later showed the tumor had disappeared, so we diagnosed with CR (Figure 2). Improvement was also confirmed by echocardiography. Six months after the diagnosis of right atrial metastasis, the patient continues to receive EV treatment, and the tumors are still shrinking. We showed a swimmer’s plot to illustrate the timeline and progression of bladder cancer from the initial diagnosis to the present (Figure 3).

## Discussion

Cisplatin-based regimens have been widely used as anticancer chemotherapy for bladder cancer and are still recommended as first-line treatment. In recent years, with the advent of immune checkpoint inhibitors, options such as second-line and switch-maintenance therapies have become available. Furthermore, in 2021, EV was approved for insurance coverage in Japan, since it has mainly been administered as a subsequent treatment following immune checkpoint inhibitor therapy. EV, an antibody-drug conjugate, consists of a human monoclonal antibody against nectin-4 and the microtubule-disrupting agent monomethyl auristatin E, which causes cell cycle arrest and cell death. The EV-301 trial demonstrated a survival benefit over chemotherapy for patients with previously treated, locally advanced disease. The median overall survival was 15.18 months for EV and 10.55 months for standard chemotherapy (comprising docetaxel or paclitaxel in Japan). The median progression-free survival was 6.47 months for EV and 5.39 months for standard chemotherapy. The overall response rate was 34.4% for EV and 21.3% for standard chemotherapy [2]. Nectin-4 is expressed in the urothelium and is primarily localized to the plasma membrane or cytoplasm of tumor cells. Patients with strong nectin-4 expression reportedly tend to have better outcomes than those with weaker expression [3].

Cardiac metastases from bladder cancer are estimated to be present in 10% of autopsy cases [4,5]; clinically diagnosed cardiac metastases are even rarer. Cardiac thrombosis is one of the conditions that must be differentiated from cardiac metastasis. Echocardiograms and contrast-enhanced MRI are useful for diagnosis, and PET scans can also be considered. However, in practice, adequate testing may be difficult if the patient's condition is poor [5-8]. For these reasons, cardiac metastases were diagnosed as antemortem in fewer than half of the 31 reviewed cases. Even when such metastases are diagnosed before death, the patient’s condition is often serious, resulting in extremely poor prognosis and survival rates [9].

Hattori et al. investigated 14 patients with urothelial carcinoma with pericardial metastases and found that most died within one year [10]. We have summarized data from reports of cardiac metastases from bladder cancer. The time from diagnosis of bladder cancer to diagnosis of cardiac metastases ranged from four to 204 months (mean 48.69 months). The time from diagnosis of cardiac metastases to death ranged from five hours to 360 days (mean 60.69 days) [3]. The time from our patient’s diagnosis of primary cancer to diagnosis of cardiac metastasis was 66 months, which is close to the average reported value. However, our patient’s progress was remarkably good, with the tumor continuing to shrink after six months of ongoing treatment. One group of researchers found that, although prior treatment with pembrolizumab did not significantly affect the overall response rate to EV, it was associated with a shorter progression-free survival than after treatment with avelumab [2,11]. However, the EV-302/KEYNOTE-A39 study concluded that being an immune checkpoint inhibitor, pembrolizumab improves the tumor's immune environment [12]. Further, a superior therapeutic effect was achieved by combining it with direct tumor cell destruction by using the antibody-drug conjugate EV. EV treatment effect may vary depending on former PDL1 or PD1 antibody treatment. Our patient achieved CR after pembrolizumab treatment and showed a good outcome. Prior administration of pembrolizumab may have contributed to achieving results that are similar to those of that study.

One limitation of our report is that, because we were not able to remove or biopsy the cardiac metastases, there is no histopathological information comparison of the degree of expression of nectin-4 in the cardiac metastases with that in the primary site. The degree of expression of nectin-4 has the potential to become an important predictor of the effect of EV. Despite this limitation, we here report a patient with right atrial metastasis from bladder cancer in whom treatment with EV achieved a preferable therapeutic effect.

## Conclusions

Right atrial metastasis following total cystectomy is an uncommon occurrence and is generally associated with a poor prognosis. In our case, we report the first instance of complete tumor remission in the right atrium achieved through EV therapy, with the remission persisting for six months. These findings suggest that EV treatment holds significant potential, even in cases involving cardiac metastases, and could serve as a promising cornerstone of therapeutic strategies for both patients and clinicians.
